# TISIGNER.com: web services for improving recombinant protein production

**DOI:** 10.1093/nar/gkab175

**Published:** 2021-03-21

**Authors:** Bikash K Bhandari, Chun Shen Lim, Paul P Gardner

**Affiliations:** Department of Biochemistry, School of Biomedical Sciences, University of Otago, Dunedin 9054, New Zealand; Department of Biochemistry, School of Biomedical Sciences, University of Otago, Dunedin 9054, New Zealand; Department of Biochemistry, School of Biomedical Sciences, University of Otago, Dunedin 9054, New Zealand; Biomolecular Interaction Centre, University of Canterbury, Christchurch 8140, New Zealand

## Abstract

Experiments that are planned using accurate prediction algorithms will mitigate failures in recombinant protein production. We have developed TISIGNER (https://tisigner.com) with the aim of addressing technical challenges to recombinant protein production. We offer three web services, TIsigner (Translation Initiation coding region designer), SoDoPE (Soluble Domain for Protein Expression) and Razor, which are specialised in synonymous optimisation of recombinant protein expression, solubility and signal peptide analysis, respectively. Importantly, TIsigner, SoDoPE and Razor are linked, which allows users to switch between the tools when optimising genes of interest.

## Graphical Abstract

**Graphical Abstract:**
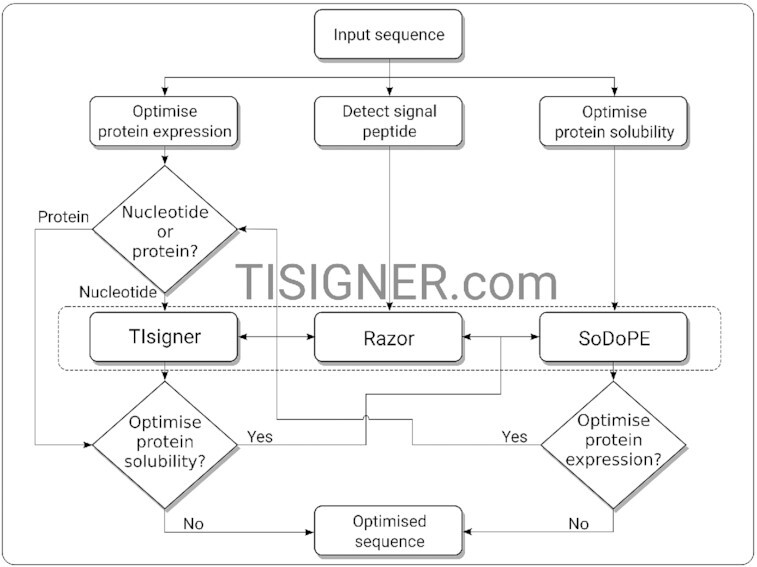
Optimising recombinant protein production using TISIGNER.com

## INTRODUCTION

Recombinant protein production is a key process for life science research and the development of biotherapeutics. However, low protein expression and aggregation are the two major bottlenecks of recombinant protein production (1–7). Since mRNA abundance alone is insufficient to explain protein abundance (8–12), several features of mRNA sequence have been proposed to affect protein expression. These features are mostly related to codon usage, such as the codon adaptation index and tRNA adaptation index (13–17), or measures of mRNA secondary structure, such as G+C content, minimum free energy (MFE) of RNA secondary structure, and mRNA:ncRNA interaction avoidance (18–23). Many of these features are not independent, making it challenging to distinguish the impacts of individual features (24). This, in turn, hinders the development of accurate prediction/optimisation tools. Recent systematic studies suggest that MFE is the most important feature in protein expression (24,25). However, more recent work shows that the mRNA accessibility of translation initiation sites outperforms MFE in predicting relative protein levels from mRNA sequences (26,27). Accessibility is computed by considering all possible structures for a region, weighted by free energy, not just the single structure with the MFE (28).

In addition to high protein expression level, high solubility is preferable for the purification and long-term storage of recombinant proteins. However, almost half of the successfully expressed proteins are insoluble (http://targetdb.rcsb.org/metrics), which makes the recombinant protein production process challenging. A number of methods have been suggested to improve protein solubility, for example, truncation, mutagenesis, and the use of solubility-enhancing tags (2,29–31). Nevertheless, accurate solubility prediction could save resources and aid in designing soluble proteins before the experiments. With these in mind, we have recently formulated the solubility-weighted index (SWI), which outperforms recent solubility prediction tools based on machine-learning algorithms (32).

Besides, many recombinant proteins of interest are secretory. The intracellular accumulation of heterologous secretory proteins may be toxic to the host cells. Therefore, the translocation efficiency of these proteins plays an important role in the yield quantity and quality. Secretory proteins usually have a short peptide at the N-terminus called signal peptide (SP), which is responsible for the translocation of secretory proteins via the Sec, signal recognition particle (SRP) or twin arginine transport (Tat) pathways (33–36). Detection of SPs or fusion of a suitable SP at the N-terminus is useful for optimising protein production (37–40). In addition, different pathways have different advantages, for example, the SRP dependent pathway can be used for rapidly folding proteins (41). However, the Sec dependent pathway, which is common across all forms of life, has been widely used for recombinant protein expression because of higher protein production capacity and quality (41,42). In addition, the presence of SPs should almost always be checked when planning the expression experiments for uncharacterised proteins.

Existing web tools predict or optimise either protein expression or solubility alone (43–51). Several web tools exist for predicting SPs (52–56). Only a very few tools can detect toxic proteins, for example, SpiderP, ClanTox and ToxinPred (57–59). These tools are either limited to predicting the venoms of certain organisms, such as spiders, or they are not designed to predict the signal peptides of toxins, rather to predict the toxicity of mature peptides. Moreover, these tools are offered through different independent services. We reasoned these functionalities should be integrated in order to assist not only in choosing appropriate expression systems, but also in optimising the expression and solubility levels of recombinant proteins. Here we present TISIGNER.com that integrates the optimisation tools TIsigner (translation initiation coding region designer), SoDoPE (soluble domain for protein expression) for protein expression and solubility, respectively, and Razor for detecting SPs (26,32,60). Our web application provides easy, fast and interactive ways to assist users in planning and designing their experiments.

## WEB SERVICES

### TIsigner

**Figure 1.**
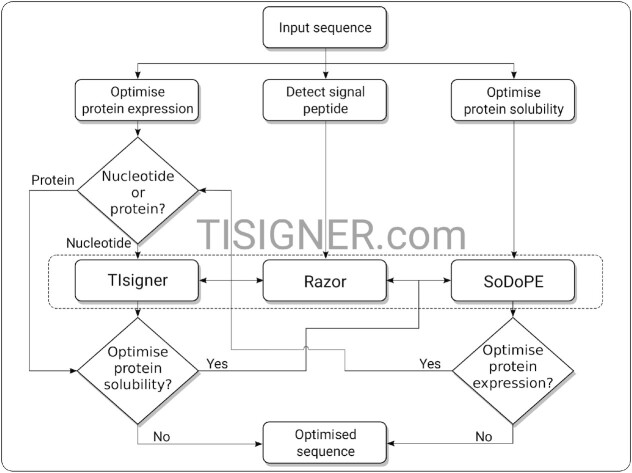
Flow chart for optimising recombinant protein production using the TISIGNER web application. TIsigner, SoDoPE and Razor are linked so that protein expression and solubility can be seamlessly optimised. TIsigner accepts a nucleotide sequence as input, whereas SoDoPE and Razor accept either a nucleotide or protein sequence. SoDoPE, soluble domain for protein expression; TIsigner, translation initiation coding region designer.

TIsigner offers tunable protein expression by optimising the mRNA accessibility of translation initiation sites (26). The regions used to calculate accessibility (opening energy) are specific to the expression hosts, which is calculated using RNAplfold (28,61,62). For *Escherichia coli*, *Saccharomyces cerevisiae*, and *Mus musculus* expression hosts, the optimal regions relative to the start codon for optimisation are −24:24, −7:89, −8:11, respectively. For other expression hosts, we provide an option ‘Other’, which optimises the accessibility of the region −24:89. Since *E. coli* is the most popular expression host, the default settings aim to optimise protein expression in *E. coli* with the T7 lac promoter system (see below). In this case, only the protein coding sequence is required for input where the 5′UTR (5′ untranslated region) sequence used as default is the most popular, truncated version of the T7 promoter (63) (Figure 1). Otherwise, the 5′UTR sequence is also required. For 5′UTRs shorter than 71 nucleotides, upstream sequences can be used to extend the UTRs.

The settings for TIsigner are grouped by complexity (i.e. general, extra, and advanced). The general settings include the options to modify the expression host, promoter and target expression score. The target expression score ranges from 0 to 100 (i.e. from the minimum to maximum predicted level), which is derived from a logistic regression of the opening energy distribution of 11 430 expression experiments in *Escherichia coli* from the ‘Protein Structure Initiative: Biology’ (PSI:Biology) (64,65). Hence, this scoring system is only applicable to the *E. coli* T7 lac promoter system. Since, there is a non-linear relationship between opening energy and expression score, an interactive plot is also displayed along with the slider to set the target expression score. For other expression hosts and promoters, the target expression level can be either maximised or minimised (i.e. binary). The extra settings have the options to optimise sequence within the translation initiation region or the full-length sequence. The AarI, BsaI, BsmBI restriction modification sites are filtered by default, whereas other sites can be manually supplied (e.g. a Shine-Dalgarno motif or terminator U-tract). The advanced settings allows users to tweak the random seed and sampling options (i.e., quick or deep, which uses different numbers of iterations and parallel processes). Here users can also customise the region for optimisation or disable the terminator checks.

**Figure 2.**
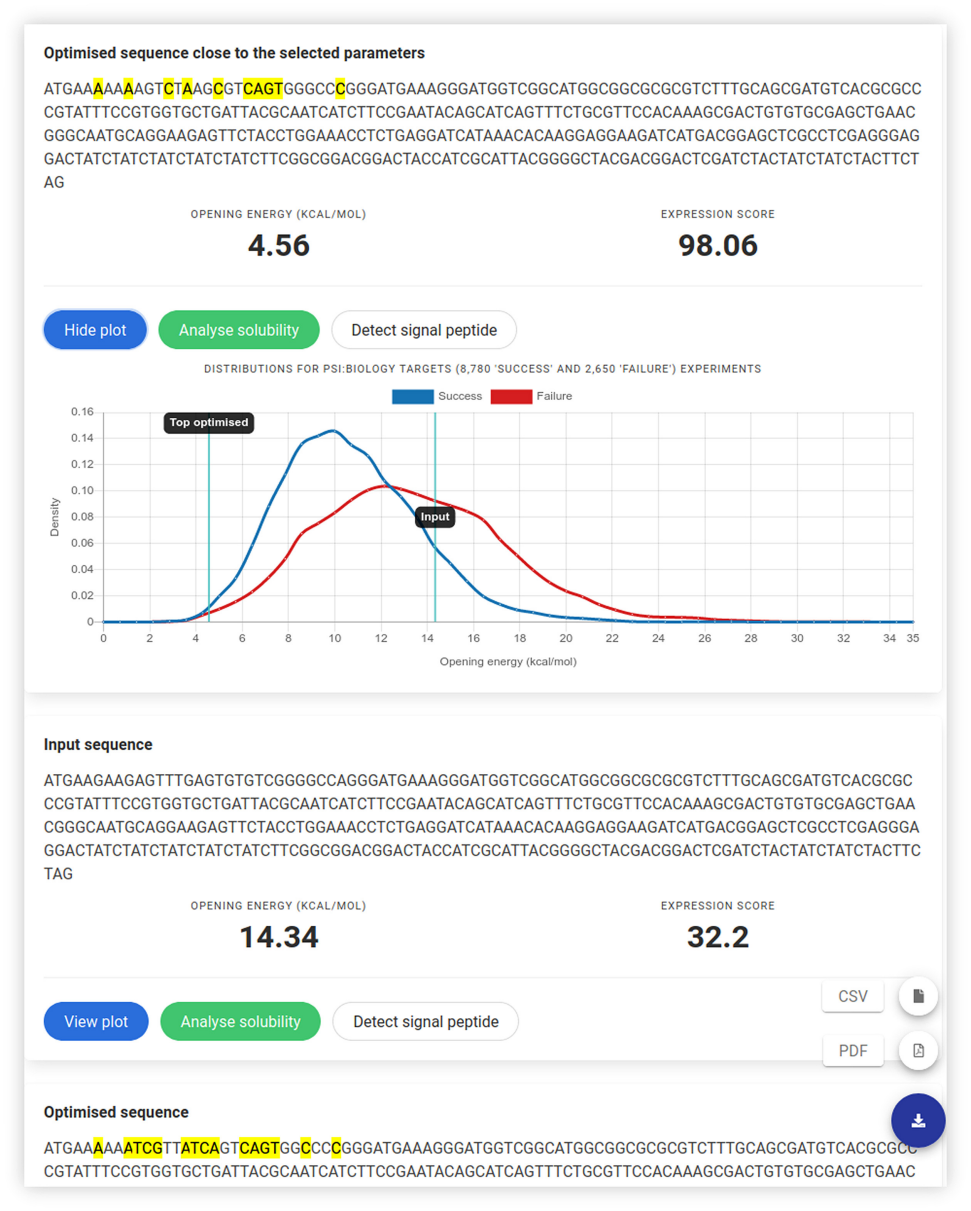
The results of TIsigner shows a protein expression optimised nucleotide sequence. The highlighted nucleotides show changes made to the input sequence. The opening energy of the input sequence before and after optimisation is annotated over the distributions of the opening energy for 8780 ‘success’ and 2650 ‘failure’ experiments from PSI:Biology. Further optimised sequences, if found, are also displayed. The results can be downloaded in either CSV or PDF format using the download icon on the bottom right. Each resulting sequence can be analysed for solubility or signal peptide.

Once the input sequence passes a sanity check, the optimisation task is rapid [*O*(1) time using RNAplfold v2.4.11 (using parameters -W 210 -u 210)] with our simulated annealing algorithm. A list of optimized sequences are returned after checking for terminators using cmsearch (Infernal v1.1.2) (66) with RMfam models (67,68). If terminators are found, an option to use the full-length sequence for optimisation will be prompted to users. In a default case (*E. coli* T7 lac promoter system), the optimised sequence closest to the chosen expression level is selected as the first solution (Figure 2). For other expression hosts and/or promoters, the optimised sequence with the minimum changes in nucleotides is selected as the first solution. The altered nucleotides are highlighted (Figure 2). The accessibility of translation initiation sites for both the input and optimised sequences is shown as opening energy (kcal/mol). The results can be exported as a PDF or CSV file. When the default settings are used, the opening energy for each sequence is indicated on the distributions of the opening energy of 8780 ‘success’ and 2650 ‘failure’ groups of the PSI:Biology target genes. Furthermore, options for solubility and SP analyses using SoDoPE and Razor, respectively, are available for each sequence on the same results page (Figure 2).

### SoDoPE

**Figure 3.**
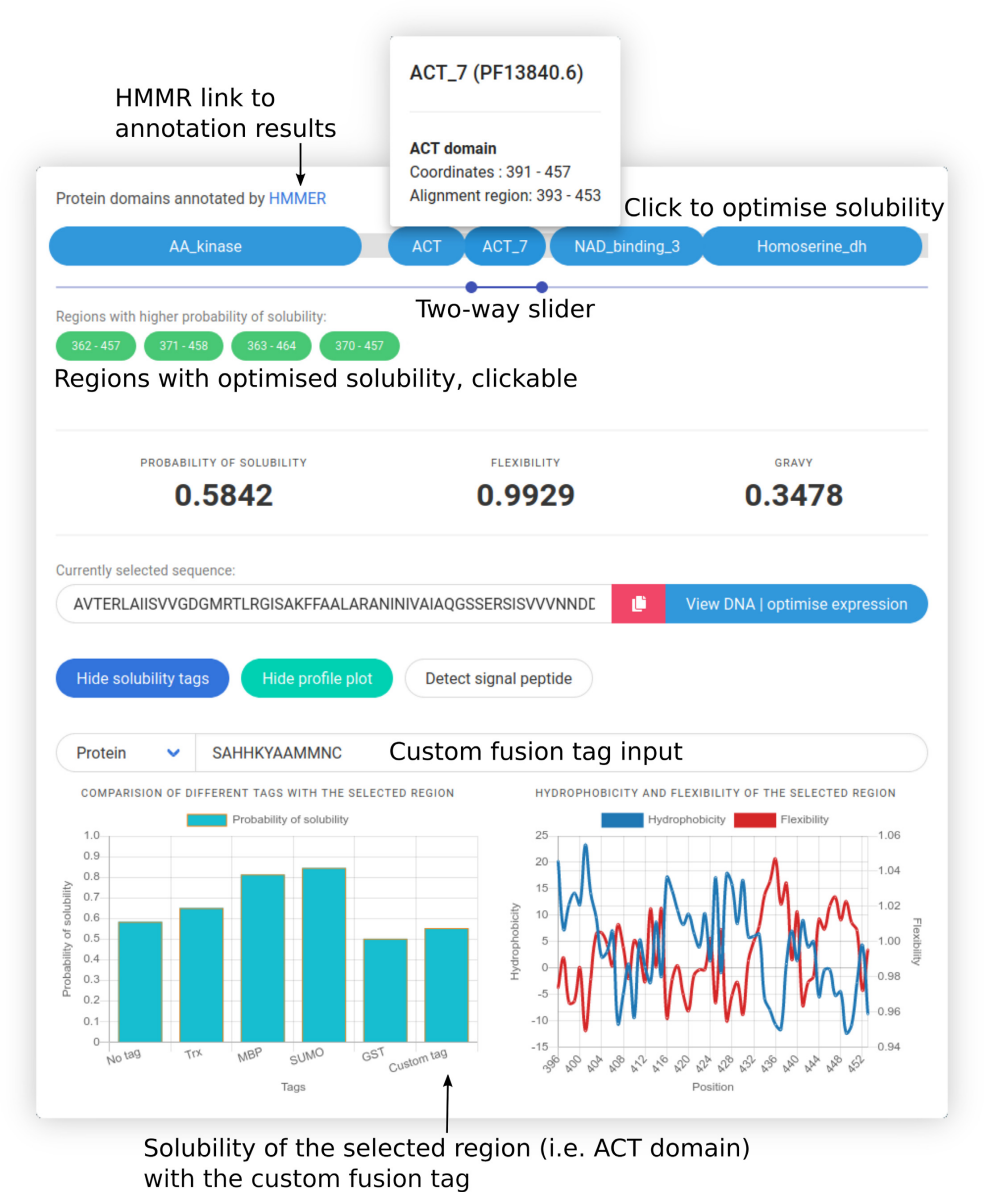
Exploring and optimizing protein solubility using SoDoPE interactive graphics. Upon clicking a protein domain or selecting a region of interest, its solubility is optimised in real-time, and a list of regions with extended boundaries and higher probabilities of solubility is returned as green buttons (clickable). The probabilities of solubility of the selected region with and without fusion tags can be visualized in a barplot. The flexibility and hydrophobicity profile plots for the selected region can also be selectively viewed. The sequence can also be checked for the presence of a signal peptide or optimized for protein expression.

SoDoPE is our interactive solubility analysis and optimisation tool based on the SWI (32). SoDoPE accepts either a nucleotide or protein sequence (Figure 1). Upon submission, a query is sent to the HMMER web service for domain annotation (69). Successful annotations are displayed as interactive graphics, in which the annotated domains are represented as discorectangles, above a grey band that represents the input protein sequence (Figure 3). Information about a protein domain is shown upon a mouse hover. The domains can be selected for solubility analysis. For a complete domain annotation report, a link to the HMMER results page is also provided.

In addition, a two-way slider is available for navigation through any region of interest (Figure 3). The probability of solubility, flexibility and GRAVY (grand average of hydropathicity) is shown in real-time according to the user-selected region. The selected region is optimised for higher solubility using simulated annealing. Only the regions with extended boundaries and also higher probability of solubility is returned. SP analysis can also be done using Razor (see below).

A profile plot of flexibility and/or hydrophilicity corresponding to the user selected region is generated (Figure 3). This allows an estimation of rigid/flexible regions and possible helices, that may be helpful for mutagenesis experiments. The sequence of the selected region is shown, with the option of sequence conversion between nucleotide and amino acid sequence format. In particular, the nucleotide sequence can be redirected to TIsigner for optimising protein expression (Figures 1 and 3, through the ‘view DNA | optimise expression’ button).

The contributions of several solubility-enhancing tags to user selected regions can be compared and shown in a bar plot, including thioredoxin (TRX), maltose binding protein (MBP), small ubiquitin-related modifier (SUMO) and glutathione-*S*-transferase (GST) tags (Figure 3). Users can also input a fusion sequence of interest either in a nucleotide or protein sequence format.

### Razor

**Figure 4.**
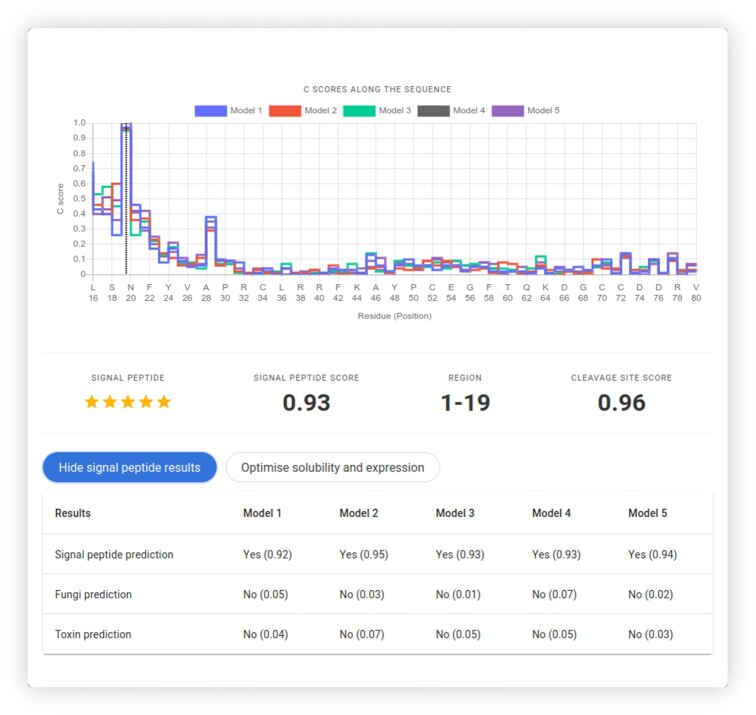
Detection of signal peptides using Razor. The dotted annotation in the step plot for the cleavage site scores (*C*-scores) shows the most likely position for proteolytic cleavage. The sequence can also be checked and optimised for protein solubility and expression.

Razor is our SP prediction tool which is based upon random forest models of protein features from the eukaryotic SP sequences of the SignalP 5.0 dataset and the animal toxin annotation project (52,60,70). Razor accepts either a protein or a nucleotide sequence (Figure 1). After validation, the N-terminal region is checked for the presence of a SP using five random forest models. This gives five SP scores (*S*-scores) for a given sequence. For detecting the cleavage site, we use a sliding window of 30 residues and our optimised weight matrix for residues around the cleavage site. The scored subsequences are scored by additional five random forest models to give the cleavage site scores (*C*-scores) along the sequence, which is displayed as a step plot (Figure 4). The *Y*-score, which is the geometric mean of *S*-scores and the max of *C*-scores, is used to infer whether the given sequence has a SP or not. The median of these five *Y*-scores is displayed as the final score. The cleavage site from the model with the median of max of *C*-scores is used to annotate the predicted region.

If any of the models detect a SP in the input sequence, we further check whether the SP belongs to toxins, using five random forests trained on toxin SPs. The final toxin score is the median of scores from those random forest models. Furthermore, since we noticed a lack of tools specialising in predicting SPs from fungi, any detected signal peptide is checked for such origin. Similarly, we use five random forests for detecting fungal SPs, with the final fungal score being the median score of these models. Since we have five random forest models in each step (eukaryotic, toxin and fungal SP detection steps), stars are displayed as an indication of the number of models agreeing on the sequence falling on either category (Figure 4).

Razor is linked with SoDoPE for checking and optimising protein solubility (Figure 4). If a nucleotide sequence was submitted, this sequence can also be optimised for protein expression using TIsigner (Figure 1).

## DISCUSSION

Low protein expression and solubility are the major hindrances to a successful recombinant protein production. Based on our comprehensive studies on these two problems, we have developed novel tools to optimise protein expression (TIsigner) and solubility (SoDoPE), and assessed their predictive performance using independent datasets (Supplementary Table S1). Our tools offer some unique features in an interactive way. TIsigner allows tuning of protein expression from low to high levels, whereas SoDoPE allows easy navigation of protein sequence/domains with real-time solubility prediction. Based on our assessment of similar tools, none of the publicly available tools provides these features.

Our third tool, Razor, is designed to check the presence of SPs. Compared to other related tools, Razor also predicts toxin and fungal SPs (Supplementary Table S2). These would be helpful for users in choosing the expression and purification systems that prevent the harmful intracellular accumulation of recombinant secretory proteins/toxins.

Our tools are interactive, fast, and accurate. Importantly, our tools are highly integrated, allowing a seamless transition between the optimisation tools. To make such transition intuitive, our web services limits one input sequence at a time and we aim to remove this input sequence limitation in the future. For optimising a large number of sequence, we provide the command-line version of each of our tools (see below).

## GENERAL INFORMATION

Demo input and results are available for new users to get started. A list of frequently asked questions is also available for each tool. The frontend is written in React and uses responsive web design principles. The backend is written in Flask and Python v3.6. The website is hosted on a virtual machine (Red Hat Enterprise Linux 8) running on Intel Xeon (8 × 2.60 GHz) with 4GiB RAM, by the Information Technology Services at the University of Otago.

## DATA AVAILABILITY

The web server is available at https://tisigner.com. This website is free and open to all users and there is no login required. All our tools, and the website are open-sourced (https://github.com/Gardner-BinfLab/TISIGNER-ReactJS; https://github.com/Gardner-BinfLab/TIsigner/tree/master/TIsigner_cmd; https://github.com/Gardner-BinfLab/SoDoPE_paper_2020/tree/master/SWI; https://github.com/Gardner-BinfLab/Razor) and privacy friendly (no data stored).

## Supplementary Material

gkab175_Supplemental_File

## References

[1] Berlec A. , Strukelj B. Current state and recent advances in biopharmaceutical production in *Escherichia coli*, yeasts and mammalian cells. J. Ind. Microbiol. Biotechnol. 2013; 40:257–274. 23385853 10.1007/s10295-013-1235-0

[2] Esposito D. , Chatterjee D.K. Enhancement of soluble protein expression through the use of fusion tags. Curr. Opin. Biotechnol. 2006; 17:353–358. 16781139 10.1016/j.copbio.2006.06.003

[3] Hou Q. , Bourgeas R., Pucci F., Rooman M. Computational analysis of the amino acid interactions that promote or decrease protein solubility. Scientific Rep. 2018; 8:14661. 10.1038/s41598-018-32988-w PMC6168528 30279585

[4] Kramer R.M. , Shende V.R., Motl N., Pace C.N., Scholtz J.M. Toward a molecular understanding of protein solubility: increased negative surface charge correlates with increased solubility. Biophys. J. 2012; 102:1907–1915. 22768947 10.1016/j.bpj.2012.01.060 PMC3328702

[5] Mazurenko S. Predicting protein stability and solubility changes upon mutations: data perspective. ChemCatChem. 2020; 12:5590–5598.

[6] Rosano G.L. , Ceccarelli E.A. Recombinant protein expression in *Escherichia coli*: advances and challenges. Front. Microbiol. 2014; 5:172. 24860555 10.3389/fmicb.2014.00172 PMC4029002

[7] Vihinen M. Solubility of proteins. ADMET DMPK. 2020; 8:391–399. 10.5599/admet.831 PMC8915590 35300195

[8] Bernstein J.A. , Khodursky A.B., Lin P.-H., Lin-Chao S., Cohen S.N. Global analysis of mRNA decay and abundance in *Escherichia coli* at single-gene resolution using two-color fluorescent DNA microarrays. Proc. Natl. Acad. Sci. U.S.A. 2002; 99:9697–9702. 12119387 10.1073/pnas.112318199 PMC124983

[9] de Sousa Abreu R. , Penalva L.O., Marcotte E.M., Vogel C. Global signatures of protein and mRNA expression levels. Mol. Biosyst. 2009; 5:1512–1526. 20023718 10.1039/b908315d PMC4089977

[10] Lim C.S. , T. Wardell S.J., Kleffmann T., Brown C.M. The exon–intron gene structure upstream of the initiation codon predicts translation efficiency. Nucleic Acids Res. 2018; 46:4575–4591. 29684192 10.1093/nar/gky282 PMC5961209

[11] Nieuwkoop T. , Finger-Bou M., van der Oost J., Claassens N.J. The ongoing quest to crack the genetic code for protein production. Mol. Cell. 2020; 80:193–209. 33010203 10.1016/j.molcel.2020.09.014

[12] Taniguchi Y. , Choi P.J., Li G.-W., Chen H., Babu M., Hearn J., Emili A., Xie X.S. Quantifying *E. coli* proteome and transcriptome with single-molecule sensitivity in single cells. Science. 2010; 329:533–538. 20671182 10.1126/science.1188308 PMC2922915

[13] Brule C.E. , Grayhack E.J. Synonymous codons: choose wisely for expression. Trends Genet. 2017; 33:283–297. 28292534 10.1016/j.tig.2017.02.001 PMC5409834

[14] dos Reis M. , Savva R., Wernisch L. Solving the riddle of codon usage preferences: a test for translational selection. Nucleic Acids Res. 2004; 32:5036–5044. 15448185 10.1093/nar/gkh834 PMC521650

[15] Gutman G.A. , Hatfield G.W. Nonrandom utilization of codon pairs in *Escherichia coli*. Proc. Natl. Acad. Sci. U.S.A. 1989; 86:3699–3703. 2657727 10.1073/pnas.86.10.3699 PMC287207

[16] Sabi R. , Tuller T. Modelling the efficiency of codon–tRNA interactions based on codon usage bias. DNA Res. 2014; 21:511–526. 24906480 10.1093/dnares/dsu017 PMC4195497

[17] Sharp P.M. , Li W.H. The codon Adaptation Index–a measure of directional synonymous codon usage bias, and its potential applications. Nucleic Acids Res. 1987; 15:1281–1295. 3547335 10.1093/nar/15.3.1281 PMC340524

[18] de Smit M.H. , van Duin J. Secondary structure of the ribosome binding site determines translational efficiency: a quantitative analysis. Proc. Natl. Acad. Sci. U.S.A. 1990; 87:7668–7672. 2217199 10.1073/pnas.87.19.7668 PMC54809

[19] Dvir S. , Velten L., Sharon E., Zeevi D., Carey L.B., Weinberger A., Segal E. Deciphering the rules by which 5′-UTR sequences affect protein expression in yeast. Proc. Natl. Acad. Sci. U.S.A. 2013; 110:E2792–E2801. 23832786 10.1073/pnas.1222534110 PMC3725075

[20] Kudla G. , Murray A.W., Tollervey D., Plotkin J.B. Coding-sequence determinants of gene expression in *Escherichia coli*. Science. 2009; 324:255–258. 19359587 10.1126/science.1170160 PMC3902468

[21] Plotkin J.B. , Kudla G. Synonymous but not the same: the causes and consequences of codon bias. Nat. Rev. Genet. 2011; 12:32–42. 21102527 10.1038/nrg2899 PMC3074964

[22] Tuller T. , Zur H. Multiple roles of the coding sequence 5′ end in gene expression regulation. Nucleic Acids Res. 2015; 43:13–28. 25505165 10.1093/nar/gku1313 PMC4288200

[23] Umu S.U. , Poole A.M., Dobson R.C., Gardner P.P. Avoidance of stochastic RNA interactions can be harnessed to control protein expression levels in bacteria and archaea. Elife. 2016; 5:e13479. 27642845 10.7554/eLife.13479 PMC5028192

[24] Mauger D.M. , Cabral B.J., Presnyak V., Su S.V., Reid D.W., Goodman B., Link K., Khatwani N., Reynders J., Moore M.J. et al. mRNA structure regulates protein expression through changes in functional half-life. Proc. Natl. Acad. Sci. U.S.A. 2019; 116:24075–24083. 31712433 10.1073/pnas.1908052116 PMC6883848

[25] Cambray G. , Guimaraes J.C., Arkin A.P. Evaluation of 244,000 synthetic sequences reveals design principles to optimize translation in *Escherichia coli*. Nat. Biotechnol. 2018; 36:1005–1015. 30247489 10.1038/nbt.4238

[26] Bhandari B.K. , Lim C.S., Gardner P.P. Protein yield is tunable by synonymous codon changes of translation initiation sites. 2021; bioRxiv doi:22 February 2021, preprint: not peer reviewed 10.1101/726752. PMC8519471 34610008

[27] Terai G. , Asai K. Improving the prediction accuracy of protein abundance in *Escherichia coli* using mRNA accessibility. Nucleic Acids Res. 2020; 48:e81. 32504488 10.1093/nar/gkaa481 PMC7641306

[28] Bernhart S.H. , Hofacker I.L., Stadler P.F. Local RNA base pairing probabilities in large sequences. Bioinformatics. 2006; 22:614–615. 16368769 10.1093/bioinformatics/btk014

[29] Chan W.-C. , Liang P.-H., Shih Y.-P., Yang U.-C., Lin W.-C., Hsu C.-N. Learning to predict expression efficacy of vectors in recombinant protein production. BMC Bioinformatics. 2010; 11:S21. 10.1186/1471-2105-11-S1-S21 PMC3009492 20122193

[30] Costa S. , Almeida A., Castro A., Domingues L. Fusion tags for protein solubility, purification and immunogenicity in *Escherichia coli*: the novel Fh8 system. Front. Microbiol. 2014; 5:63. 24600443 10.3389/fmicb.2014.00063 PMC3928792

[31] Waldo G.S. Genetic screens and directed evolution for protein solubility. Curr. Opin. Chem. Biol. 2003; 7:33–38. 12547424 10.1016/s1367-5931(02)00017-0

[32] Bhandari B.K. , Gardner P.P., Lim C.S. Solubility-weighted index: fast and accurate prediction of protein solubility. Bioinformatics. 2020; 36:4691–4698. 32559287 10.1093/bioinformatics/btaa578 PMC7750957

[33] Luirink J. , Dobberstein B. Mammalian and *Escherichia coli* signal recognition particles. Mol. Microbiol. 1994; 11:9–13. 8145649 10.1111/j.1365-2958.1994.tb00284.x

[34] Palmer T. , Berks B.C. The twin-arginine translocation (Tat) protein export pathway. Nat. Rev. Microbiol. 2012; 10:483–496. 22683878 10.1038/nrmicro2814

[35] Rusch S.L. , Kendall D.A. Interactions that drive Sec-dependent bacterial protein transport. Biochemistry. 2007; 46:9665–9673. 17676771 10.1021/bi7010064 PMC2675607

[36] von Heijne G. The signal peptide. J. Membr. Biol. 1990; 115:195–201. 2197415 10.1007/BF01868635

[37] Freudl R. Signal peptides for recombinant protein secretion in bacterial expression systems. Microb. Cell Fact. 2018; 17:52. 29598818 10.1186/s12934-018-0901-3 PMC5875014

[38] Karyolaimos A. , Dolata K.M., Antelo-Varela M., Borras A.M., Elfageih R., Sievers S., Becher D., Riedel K., de Gier J.-W. *Escherichia coli* can adapt its protein translocation machinery for enhanced periplasmic recombinant protein production. Front. Bioeng. Biotechnol. 2020; 7:465. 32064253 10.3389/fbioe.2019.00465 PMC7000420

[39] Rosano G.L. , Morales E.S., Ceccarelli E.A. New tools for recombinant protein production in *Escherichia coli*: A 5-year update. Protein Sci. 2019; 28:1412–1422. 31219641 10.1002/pro.3668 PMC6635841

[40] Zamani M. , Nezafat N., Negahdaripour M., Dabbagh F., Ghasemi Y. *In Silico* evaluation of different signal peptides for the secretory production of human growth hormone in *E. coli*. Int. J. Peptide Res. Ther. 2015; 21:261–268.

[41] Owji H. , Nezafat N., Negahdaripour M., Hajiebrahimi A., Ghasemi Y. A comprehensive review of signal peptides: structure, roles, and applications. Eur. J. Cell Biol. 2018; 97:422–441. 29958716 10.1016/j.ejcb.2018.06.003

[42] Ma R.J. , Wang Y.H., Liu L., Bai L.L., Ban R. Production enhancement of the extracellular lipase LipA in *Bacillus subtilis*: effects of expression system and Sec pathway components. Protein Expression Purif. 2018; 142:81–87. 10.1016/j.pep.2017.09.011 28963005

[43] Agostini F. , Cirillo D., Livi C.M., Delli Ponti R., Tartaglia G.G. ccSOL omics: a webserver for solubility prediction of endogenous and heterologous expression in *Escherichia coli*. Bioinformatics. 2014; 30:2975–2977. 24990610 10.1093/bioinformatics/btu420 PMC4184263

[44] Chin J.X. , Chung B. K.-S., Lee D.-Y. Codon optimization OnLine (COOL): a web-based multi-objective optimization platform for synthetic gene design. Bioinformatics. 2014; 30:2210–2212. 24728853 10.1093/bioinformatics/btu192

[45] Grote A. , Hiller K., Scheer M., Münch R., Nörtemann B., Hempel D.C., Jahn D. JCat: a novel tool to adapt codon usage of a target gene to its potential expression host. Nucleic Acids Res. 2005; 33:W526–W531. 15980527 10.1093/nar/gki376 PMC1160137

[46] Puigbò P. , Guzmán E., Romeu A., Garcia-Vallvé S. OPTIMIZER: a web server for optimizing the codon usage of DNA sequences. Nucleic Acids Res. 2007; 35:W126–W131. 17439967 10.1093/nar/gkm219 PMC1933141

[47] Hebditch M. , Carballo-Amador M.A., Charonis S., Curtis R., Warwicker J. Protein-Sol: a web tool for predicting protein solubility from sequence. Bioinformatics. 2017; 33:3098–3100. 28575391 10.1093/bioinformatics/btx345 PMC5870856

[48] Hon J. , Marusiak M., Martinek T., Kunka A., Zendulka J., Bednar D., Damborsky J. SoluProt: prediction of soluble protein expression in *Escherichia coli*. Bioinformatics. 2021; doi:10.1093/bioinformatics/btaa1102. 10.1093/bioinformatics/btaa1102 PMC8034534 33416864

[49] Smialowski P. , Doose G., Torkler P., Kaufmann S., Frishman D. PROSO II–a new method for protein solubility prediction. FEBS J. 2012; 279:2192–2200. 22536855 10.1111/j.1742-4658.2012.08603.x

[50] Sormanni P. , Aprile F.A., Vendruscolo M. The CamSol method of rational design of protein mutants with enhanced solubility. J. Mol. Biol. 2015; 427:478–490. 25451785 10.1016/j.jmb.2014.09.026

[51] Zayni S. , Damiati S., Moreno-Flores S., Amman F., Hofacker I., Ehmoser E.-K. Enhancing the cell-free expression of native membrane proteins by in-silico optimization of the coding sequence – an experimental study of the human voltage-dependent anion channel. 2018; bioRxiv doi:07 September 2018, preprint: not peer reviewed 10.1101/411694. PMC8540592 34677509

[52] Almagro Armenteros J.J. , Tsirigos K.D., Sønderby C.K., Petersen T.N., Winther O., Brunak S., von Heijne G., Nielsen H. SignalP 5.0 improves signal peptide predictions using deep neural networks. Nat. Biotechnol. 2019; 37:420–423. 30778233 10.1038/s41587-019-0036-z

[53] Bagos P.G. , Tsirigos K.D., Plessas S.K., Liakopoulos T.D., Hamodrakas S.J. Prediction of signal peptides in archaea. Protein Eng. Des. Sel. 2009; 22:27–35. 18988691 10.1093/protein/gzn064

[54] Hiller K. , Grote A., Scheer M., Münch R., Jahn D. PrediSi: prediction of signal peptides and their cleavage positions. Nucleic Acids Res. 2004; 32:W375–W379. 15215414 10.1093/nar/gkh378 PMC441516

[55] Käll L. , Krogh A., Sonnhammer E. L.L. A combined transmembrane topology and signal peptide prediction method. J. Mol. Biol. 2004; 338:1027–1036. 15111065 10.1016/j.jmb.2004.03.016

[56] Savojardo C. , Martelli P.L., Fariselli P., Casadio R. DeepSig: deep learning improves signal peptide detection in proteins. Bioinformatics. 2017; 34:1690–1696. 10.1093/bioinformatics/btx818 PMC5946842 29280997

[57] Gupta S. , Kapoor P., Chaudhary K., Gautam A., Kumar R. Open Source Drug Discovery Consortium and Raghava G. P.S. *In silico* approach for predicting toxicity of peptides and proteins. PLoS One. 2013; 8:e73957. 24058508 10.1371/journal.pone.0073957 PMC3772798

[58] Naamati G. , Askenazi M., Linial M. ClanTox: a classifier of short animal toxins. Nucleic Acids Res. 2009; 37:W363–W368. 19429697 10.1093/nar/gkp299 PMC2703885

[59] Wong E. S.W. , Hardy M.C., Wood D., Bailey T., King G.F. SVM-based prediction of propeptide cleavage sites in spider toxins identifies toxin innovation in an Australian tarantula. PLoS One. 2013; 8:e66279. 23894279 10.1371/journal.pone.0066279 PMC3718798

[60] Bhandari B.K. , Gardner P.P., Lim C.S. Razor: annotation of signal peptides from toxins. 2021; bioRxiv doi:07 March 2021, preprint: not peer reviewed 10.1101/2020.11.30.405613.

[61] Bernhart S.H. , Mückstein U., Hofacker I.L. RNA accessibility in cubic time. Algorithms Mol. Biol. 2011; 6:3. 21388531 10.1186/1748-7188-6-3 PMC3063221

[62] Lorenz R. , Bernhart S.H., Höner Zu Siederdissen C., Tafer H., Flamm C., Stadler P.F., Hofacker I.L. ViennaRNA Package 2.0. Algorithms Mol. Biol. 2011; 6:26. 22115189 10.1186/1748-7188-6-26 PMC3319429

[63] Shilling P.J. , Mirzadeh K., Cumming A.J., Widesheim M., Köck Z., Daley D.O. Improved designs for pET expression plasmids increase protein production yield in *Escherichia coli*. Commun. Biol. 2020; 3:214. 32382055 10.1038/s42003-020-0939-8 PMC7205610

[64] Chen L. , Oughtred R., Berman H.M., Westbrook J. TargetDB: a target registration database for structural genomics projects. Bioinformatics. 2004; 20:2860–2862. 15130928 10.1093/bioinformatics/bth300

[65] Seiler C.Y. , Park J.G., Sharma A., Hunter P., Surapaneni P., Sedillo C., Field J., Algar R., Price A., Steel J. et al. DNASU plasmid and PSI:Biology-Materials repositories: resources to accelerate biological research. Nucleic Acids Res. 2014; 42:D1253–D1260. 24225319 10.1093/nar/gkt1060 PMC3964992

[66] Nawrocki E.P. , Eddy S.R. Infernal 1.1: 100-fold faster RNA homology searches. Bioinformatics. 2013; 29:2933–2935. 24008419 10.1093/bioinformatics/btt509 PMC3810854

[67] Gardner P.P. , Eldai H. Annotating RNA motifs in sequences and alignments. Nucleic Acids Res. 2015; 43:691–698. 25520192 10.1093/nar/gku1327 PMC4333381

[68] Kalvari I. , Nawrocki E.P., Ontiveros-Palacios N., Argasinska J., Lamkiewicz K., Marz M., Griffiths-Jones S., Toffano-Nioche C., Gautheret D., Weinberg Z. et al. Rfam 14: expanded coverage of metagenomic, viral and microRNA families. Nucleic Acids Res. 2021; 49:D192–D200. 33211869 10.1093/nar/gkaa1047 PMC7779021

[69] Potter S.C. , Luciani A., Eddy S.R., Park Y., Lopez R., Finn R.D. HMMER web server: 2018 update. Nucleic Acids Res. 2018; 46:W200–W204. 29905871 10.1093/nar/gky448 PMC6030962

[70] Jungo F. , Bougueleret L., Xenarios I., Poux S. The UniProtKB/Swiss-Prot Tox-Prot program: a central hub of integrated venom protein data. Toxicon. 2012; 60:551–557. 22465017 10.1016/j.toxicon.2012.03.010 PMC3393831

