# Supplementary material for: TISIGNER.com: web services for improving recombinant protein production
Source: Nucleic Acids Res. 2021 Mar 21;49(W1):W654–61. doi: 10.1093/nar/gkab175 (PMC8265118; doi:10.1093/nar/gkab175)
Supplement: gkab175_Supplemental_File [file gkab175_supplemental_file.pdf]

SUPPLEMENTARY MATERIALS

Table S1. Performance metrics of TIsigner and SoDoPE (1, 2).

Tool (approach)	Dataset, host (references)	Type	Sample size	Spearman's correlation	AUROC
TIsigner (mRNA accessibility)	GFP reporter, *Escherichia coli* (3)	Continuous	14,425	−0.65[a]	N/A
	YFP reporter, *Saccharomyces cerevisiae (4)*	Continuous	2,041	-0.55[a]	N/A
	GFP reporter, *Mus musculus* (5)	Continuous	65,536	-0.28[a]	N/A
	PSI:Biology, *E. coli* (6)	Binary	8,780 expressed, 2,650 non-expressed	N/A	0.70
SoDoPE (Solubility-Weighted Index)	Training set: PSI:Biology, *E. coli* (6)	Binary	8,238 soluble, 3,978 insoluble	N/A	0.71
	Independent test set: eSOL, *E. coli* (7)	Continuous	3,198	0.50 ($P=9.46 \times 10^{-206}$)	N/A

Table S2. Performance metrics of Razor (8).

Classifier	Dataset (references)	Sample size	MCC	AUROC	AUPRC	Cleavage site	
						Precision	Recall
Eukaryotic SP	SignalP 5.0 benchmarking set (9)	211 SPs, 7,246 non-SPs	0.815	0.98	0.85	0.565	0.597
	Independent test set	287 SPs, 52,055 non-SPs	0.405	0.96	0.61	0.136	0.596
Toxin SP	Training set (10, 11)	261 toxin SPs, 1,738 non-toxin SPs	0.741	0.89	0.74	N/A	N/A
	Independent test set (10, 11)	47 toxin SPs, 194 non-toxin SPs	0.769	0.98	0.93	N/A	N/A
Fungal SP	Training set (11)	121 fungal SPs, 1,843 non-fungal SPs	0.506	0.87	0.48	N/A	N/A
	Independent test set (11)	18 fungal SPs, 269 non-fungal SPs	0.60	0.94	0.75	N/A	N/A

[a] P-value below machine's underflow level.
AUROC and AUPRC, areas under the receiver operating characteristic curve and precision-recall curve, respectively; GFP, green fluorescent protein, MCC, Matthew's correlation coefficient, PSI:Biology, Protein Structure Initiative: Biology; YFP, yellow fluorescent protein.

References

1. Bhandari,B.K., Lim,C.S. and Gardner,P.P. (2021) Protein yield is tunable by synonymous codon changes of translation initiation sites. *bioRxiv*, doi:10.1101/726752.

2. Bhandari,B.K., Gardner,P.P. and Lim,C.S. (2020) Solubility-Weighted Index: fast and accurate prediction of protein solubility. *Bioinformatics*, **36**, 4691–4698.

3. Cambray,G., Guimaraes,J.C. and Arkin,A.P. (2018) Evaluation of 244,000 synthetic sequences reveals design principles to optimize translation in Escherichia coli. *Nat. Biotechnol.*, **36**, 1005–1015.

4. Dvir,S., Velten,L., Sharon,E., Zeevi,D., Carey,L.B., Weinberger,A. and Segal,E. (2013) Deciphering the rules by which 5'-UTR sequences affect protein expression in yeast. *Proc. Natl. Acad. Sci. U. S. A.*, **110**, E2792–801.

5. Noderer,W.L., Flockhart,R.J., Bhaduri,A., Diaz de Arce,A.J., Zhang,J., Khavari,P.A. and Wang,C.L. (2014) Quantitative analysis of mammalian translation initiation sites by FACS-seq. *Mol. Syst. Biol.*, **10**, 748.

6. Seiler,C.Y., Park,J.G., Sharma,A., Hunter,P., Surapaneni,P., Sedillo,C., Field,J., Algar,R., Price,A., Steel,J., *et al.* (2014) DNASU plasmid and PSI:Biology-Materials repositories: resources to accelerate biological research. *Nucleic Acids Res.*, **42**, D1253–60.

7. Niwa,T., Ying,B.-W., Saito,K., Jin,W., Takada,S., Ueda,T. and Taguchi,H. (2009) Bimodal protein solubility distribution revealed by an aggregation analysis of the entire ensemble of Escherichia coli proteins. *Proc. Natl. Acad. Sci. U. S. A.*, **106**, 4201–4206.

8. Bhandari,B.K., Gardner,P.P. and Lim,C.S. (2021) Razor: annotation of signal peptides from toxins. *bioRxiv*, doi:10.1101/2020.11.30.405613.

9. Almagro Armenteros,J.J., Tsirigos,K.D., Sønderby,C.K., Petersen,T.N., Winther,O., Brunak,S., von Heijne,G. and Nielsen,H. (2019) SignalP 5.0 improves signal peptide predictions using deep neural networks. *Nat. Biotechnol.*, **37**, 420–423.

10. Jungo,F., Bougueleret,L., Xenarios,I. and Poux,S. (2012) The UniProtKB/Swiss-Prot Tox-Prot program: A central hub of integrated venom protein data. *Toxicon*, **60**, 551–557.

11. UniProt Consortium (2019) UniProt: a worldwide hub of protein knowledge. *Nucleic Acids Res.*, **47**, D506–D515.
